# Soft and hard tissue evaluation for vestibular socket therapy of immediately placed implants in infected and non-infected sockets: a 1-year prospective cohort study

**DOI:** 10.1186/s12903-024-04905-3

**Published:** 2024-10-07

**Authors:** Abdelsalam Elaskary, Abdelrahman Thabet, Mai Hussin, Iman Abd-ElWahab Radi

**Affiliations:** 1 Private Practice, Alexandria, Egypt; 2 Endodontology Department, Faculty of Dentistry, Alexandria University, Alexandria, Egypt; 3 Head of the Research Department, Ministry of Health, Alexandria, Egypt; 4 Professor of Prosthodontics, Faculty of Dentistry, Cairo University; Vice dean of School of Dentistry, Badya University; Member of Evidence-Based Dentistry Center, Faculty of Dentistry, Cairo University, 11 ElSaraya St, EL Manial, Cairo, 11553 Egypt

**Keywords:** Vestibular socket therapy, Immediate placement, Soft tissue level, Bone graft, Type II sockets, Guided bone regeneration, Infected sockets

## Abstract

**Background:**

Immediate implant placement using vestibular socket therapy (VST) proved to offer a successful treatment option in compromised sockets. However, the presence of active signs infection complicates immediate implants in sockets with defective labial plates, due to the possible contamination of the implant or the bone graft with existing infected tissues or oral environment via the fistula. This study, therefore, aims to explore the success of immediate implant placement using VST in managing infected compromised sockets.

**Methods:**

We included 26 age- and sex-matched patients with 41 implants sites. Thirteen patients had 19 infected (group I) and 13 had 21 non-infected type 2 sockets (group N). Both groups were treated using vestibular socket therapy (VST) and a 6-day protocol. Implant survival, changes in facial bone thickness, and mid, mesial, and distal mucosal levels were evaluated 1 year after implant placement. The Mann–Whitney U test was used to compare both groups. Furthermore, the Wilcoxon signed-rank test was used to study changes with time within each group. The statistical significance level was set at *P* < 0.05.

**Results:**

All implants survived; no significant difference was found between groups N and I regarding apical, mid, and crestal bone thickness and soft tissue level, except at the mesial papilla, where the recession was significantly more in group N than in group I. Changes over time were statistically significant in the apical, mid, and crestal bone thickness in both groups. The mean bone thickness gain ranged from 0.85 to 2.4 mm and 0.26–1.63 mm in groups I and N, respectively. Additionally, the mean mucosal recession ranged from 0.29 to 0.51 mm and 0.39–1.47 mm in groups I and N, respectively.

**Conclusion:**

Within the limitations of this study immediate implant placement in type II infected sockets using the 6-day protocol and VST achieved 100% implant survival, while maintaining the regenerated facial bone thickness with minimal mucosal recession.

**Trial registration:**

The protocol for this study was registered on clinicaltrials.gov at 3/10/2021 (registration number NCT04787224).

## Background

Immediate implant placement in fresh extraction sites, especially in the esthetic zone, is a technique sensitive procedure requiring attention to many details, including optimal implant positioning, absence of active signs of acute or chronic infection, proper management of relevant soft and hard tissues, adequate reproduction of the natural emergence profile, and meticulous accurate prosthetic steps [1]. Despite being an appealing option, immediate implant placement in compromised fresh extraction sockets is associated with many challenges, including post-extraction labial bone loss and postoperative gingival recession [2]. The latter was reported to reach up to > 1 mm in immediately placed implants compared to early implant placement, with no tissue phenotype predilection [3]. Therefore, the complexity of the situation is high in type 2 sockets, where the labial plate is defective in height, and the soft tissues are intact, but could be thin [4, 5]. The main concern with type 2 extraction sockets is not implant survival. Instead the esthetic outcome could be severely compromised, because the lack of labial bone plate increases the risk of midfacial recession and might reflect the metallic hue of the implant, especially in thin gingival phenotypes [6, 7]. Kan et al. found that the greater the defect in width, the greater risk of recession [4]. Yet, immediate implant placement has expanded by time to include compromised fresh extraction sockets with active signs of infection [8–12]. However, literature was controversial regarding the predictability of placing immediate dental implants in fresh extraction sockets with active infection. Some consider the presence of infection as a complete contraindication due to contamination and expected impaired osseointegration [13]. On the other hand, others have reported a success rate of 92–100% [14–17] using specific protocols for debriding and decontaminating the infected sockets [8–11]. Rinsing with antiseptic solutions and systemic antibiotics were reported to be beneficial for inactivating bacteria residing in inaccessible anatomically complex spaces [8]. Additionally, Kakar et al. [11] reported a survival rate of 95.45% during a mean follow-up of 48.75 ± 13.75 months for immediately placed implants in infected sockets. The authors decontaminated the sockets using laser therapy, performed in situ bone grafting for augmentation, and employed a non-submerged healing protocol.

Vestibular socket therapy (VST) is a well-studied treatment approach that enables immediate implant placement in compromised sockets, especially type 2 sockets with deficient labial plate of bone [18–20]. This technique provides a minimally invasive treatment that gives predictable esthetic outcomes and allows immediate implant placement in sockets with intact and deficient facial bone plates at follow-up period up to 2–3 years [19]. The technique also uses a “6-day protocol” that intends to disinfect sockets exhibiting active signs of infection, thereby providing optimal regenerative and esthetic results even in compromised sockets [20]. Hence, the aim of this prognostic cohort study was to compare implant survival, facial gingival recession, and bone width changes of type 2 sockets when managed by VST and “6-day protocol” in the presence and absence of infection. The null hypothesis is that there will be no significant difference between type 2 infected and non-infected sockets when managed by VST and the 6-day protocol.

## Methods

This prospective cohort study was approved by the Central Scientific Ethical Research Committee, Supreme Council of University Hospitals (approval number: NO-0311) and was reported according to Strengthening the Reporting of Observational Studies in Epidemiology (STROBE) guidelines [21]. Additionally, this study was conducted in accordance with the Declaration of Helsinki (1975), revised in 2013, the protocol of the study and was registered on clinicaltrials.gov (registration number NCT04787224, registration date 3/10/2021).

Based on the results of a systematic review [22], in which the reported risk of failure in infected sockets was 2.99 times (95% confidence interval [1.04–8.566]) that of non-infected ones, a total sample size of 34 implants, 17 per group, were required to detect a significant difference between the exposure groups at *P* < 0.05 and a power of 80%. Sample size calculation was done using GPower software (Heinrich-Heine-Universität Düsseldorf, version 3, Germany). We recruited 30 patients with 45 implant sites. The latter were frequency matched according to age and sex so that participants of infected (group I) and non-infected groups (group N) had an age range of 30–55 years. Accordingly, 26 matched patients with 41 implant sites of the recruited sample, 13 in each group, were included between January 2019 and November 2019 from a private clinical practice in Alexandria, Egypt, where implant placement and outcome assessment were performed. Clinical and radiographic examinations for all participants satisfied the following criteria: all patients were adults aged ≥ 18 years with 1–5 non-adjacent hopeless maxillary teeth in the esthetic zone. The affected teeth had type II sockets. Implants with an apical bone height, 3 mm and a primary stability < 30Ncm were excluded. Smoking and/or pregnant patients with systemic diseases and a history of chemotherapy or radiotherapy within the past 2 years were excluded. The eligible patients were informed of the nature of the study. Once they agreed to participate, they signed an informed consent form.

**Fig. 1:**
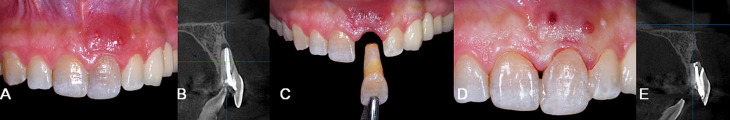
**A** Infected maxillary central incisor (Facial view); **B** CBCT scan sagittal section showing the lost labial plate of bone; **C** Reimplanting the trimmed and cleaned root of the infected tooth; **D** The reimplanted root bonded to the adjacent tooth via an acrylic resin bridge cemented to the adjacent tooth; **E** CBCT sagittal section of the reimplanted trimmed tooth

Preoperative cone beam computed tomographic (CBCT) scans were performed, the future implant position was planned, and a computer-generated surgical guide was fabricated. Despite not required in group N, the 6- day protocol [20] that mainly aims to eradicate infection, was implemented in both groups to eliminate any possibility of performance bias. In group I, signs of infection included periapical radiolucency in three sites (two patients), fistulae in two sites (two patients), sinus tracts in eleven sites (seven patients), and swelling in three sites (two patients). The 6-day protocol involved atraumatic extraction of the tooth followed by socket curettage using Elaskary irrigation curette (Stoma, Storz am Mark GmbH, Emmingen-Liptingen, Germany), mechanical debridement, and chemical irrigation using metronidazole solution (500 mg/100 mL, Amrizole, Amria Pharma, Alexandria, Egypt). The root surface of the involved tooth was cleaned using an ultrasonic cleaner, and the apical third of the root was cut off and removed. The root apex was then sealed using composite resin (Filtek™ Supreme Ultra Flowable Restorative, 3 M Corporate Headquarters, MN, USA). The root was then reimplanted into the socket stabilized to the adjacent teeth using flowable composite resin (Filtek™ Supreme Ultra Flowable Restorative, 3 M Corporate Headquarters, MN, USA) and placed totally out of the occlusion for 6 days (Fig. 1A-E).

After 6 days, the socket environment became ready for the VST protocolThe procedure started with a 1-cm long vestibular access incision that was made using a 15c blade (Stoma, Storz am Mark GmbH, Emmingen-Liptingen, Germany) at the deepest part of the vestibule opposite the socket of the involved tooth. Furthermore, a subperiosteal tunnel was created using Elaskary’s vestibular tissue elevator in a downward direction (Stoma, Storz am Mark GmbH, Emmingen-Liptingen, Germany). Implants (Biohorizons, Birmingham, Al, USA) were installed using the 3D printed surgical guide (Surgical Guide Resin, Form 2, Formlabs). A flexible cortical membrane shield (OsteoBiol® Curved Lamina, Tecnoss®, Torino, Italy) of heterologous origin, 1.0 mm in thickness, was then trimmed and tucked through the vestibular access incision until it reached 1 mm below the socket orifice and stabilized using two membrane tacs at the apical bone (AutoTac System Kit, Biohorizons Implant Systems, Birmingham, Alabama Inc, USA). The labial defect and buccal gap between the implant and shield/labial plate were then filled with particulate bone graft [50% autogenous bone chips harvested from the local surgical site and 50% dual-phase bone matrix (collagenic cortico-cancel­lous heterologous bone mix Gen-Os®, Tecnoss®, Giaveno (TO) [23].

**Fig. 2:**
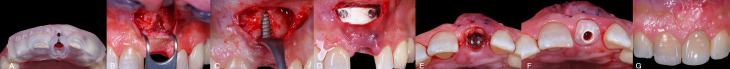
**A** Vestibular access incision and the subperiosteal tunnel created using Elaskary’s vestibular tissue elevator; **B** The 3 D printed surgical guide for computer guided implant placement; **C** Implant placed and ready to receive the bone graft; **D** The flexible cortical plate of bone fixed by a membrane tac via the vestibular access incision; **E** Soft tissue graft inserted in the socket; **F** Incisal view of the implant in place and the customized healing abutment in place; **G** Post-restorative facial view of the cement retained zirconia crown

In both groups, all sockets had thin gingival phenotype that was identified by the probe transparency test suggested by DeRouck et al. [24] As recommended by Hürzeler and Weng [25], a subepithelial connective tissue (CT) graft, harvested from the palatal tuberosity via a single incision technique was used to graft these sites. The graft was secured to the inner surface of the soft tissue rim before suturing. Finally, the vestibular incision was secured using 6/0 nylon sutures (Stoma; Storz am Mark GmbH, Emmingen-Liptingen, Germany). A temporary peek abutment (hexed PolyEtheerEtherKetone Temporary Cylinder, Biohorizons Implant Systems, Birmingham, Alabama Inc, USA) was trimmed to the socket orifice level, and the gap was sealed using composite resin (Filtek™ Supreme Ultra Flowable Restorative, 3 M Corporate Headquarters, MN, USA) to create a sealed chamber to protect the bone graft. Sutures were removed 10 days postoperatively, and the final crowns (full anatomical zirconia, bruxzir, Glidewell, CA, USA) were cemented 2 months after implant placement (Fig. 2A-G).

Patients in both groups were administered 500 mg metronidazole and 500 mg ciprofloxacin (Minapharm Pharmaceuticals, Egypt) every 12h (h), one day preoperatively, which continued for 5 days postoperatively. The antibiotic administration helped to prevent postoperative infection not only in group I, but also in group N, which had compromised labial plates of bone and hence could harbor aerobic and anaerobic bacteria. Besides, the equity in the antibiotic administration avoided any risk of performance bias. Otherwise, success in infected sockets could have been attributed to the administration of the antibiotics and not to the technique itself. Other postoperative treatment regimens included Catafast sachets 50 mg (non-steroidal anti-inflammatory drug) (Novartis, Basel, Switzerland), chlorhexidine mouthwash 0.12% (3 times daily), application of cold packs on the operative site every 20 min for the first 2h, and strict oral hygiene measures. Patients were recalled for follow-up after 1 year to check implant survival and measure gingival recession using an intra-oral scanner (IOS) (TRIOS, 3Shape A/S, Copenhagen K Denmark) and changes in bone width using CBCT.

**Fig. 3:**
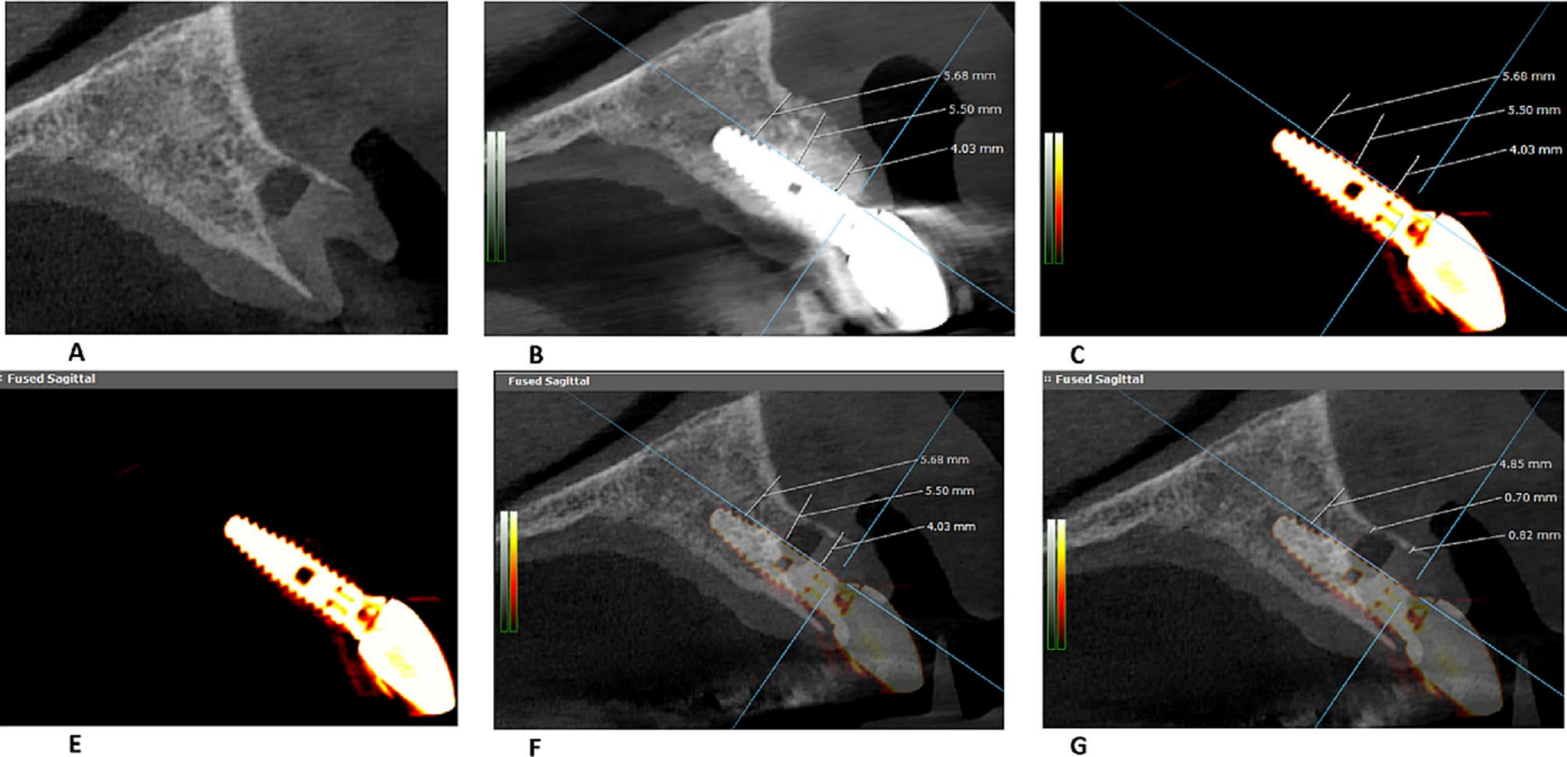
Sagittal sections of the same pre- and postoperative area to measure the bone thickness as guided by the implant; **A** The extraction socket preoperatively; **B** The operative area 2 months following implant placement showing bone thickness measurements in the center of the apical, middle and coronal regions of the implant length; **C** The operative area 2 months following implant placement after hiding the postoperative bone; **D** Implant section without the measurements; **E** Sagittal section in the implant superimposed on the preoperative bone with the postoperative measurements still on; **F** Measurements of the bone thickness preoperatively guided by the implant regions

The outcomes were recorded by two blinded, independent, and experienced examiners (ME and AA), and the average was recorded. Implant failure was reported as defined by Buser et al. [26]; the presence of peri-implant infection, persistent subjective complaints, such as pain, foreign body sensation, dysesthesia, radiolucency around the implant, and/or any detectable implant mobility. Changes in the thickness of the labial plate of the bone were measured by superimposing CBCT images obtained at baseline (time of tooth extraction and implant placement) and after 12 months. The imaging protocol was standardized by radiographing the patients with a wax interocclusal record to separate the maxillary and mandibular teeth at 5–10 Kilovoltage peak (KVp). The field of vision used was 6 × 8 pixels. Furthermore, the CBCT machine (Carestream Health, CS 8100 3D System) used for imaging had a high-contrast resolution detector (high bit depth). These specifications reduced the beam-hardening effect. In addition, the patient’s teeth helped superimpose and align the Dicom images. The difference between the two images was measured at three levels: the implant platform, half of its length, and its apex (Fig. 3) using Planmeca Romexis (viewing and planning software, version 4.6.2. R, Finland).

**Fig. 4:**
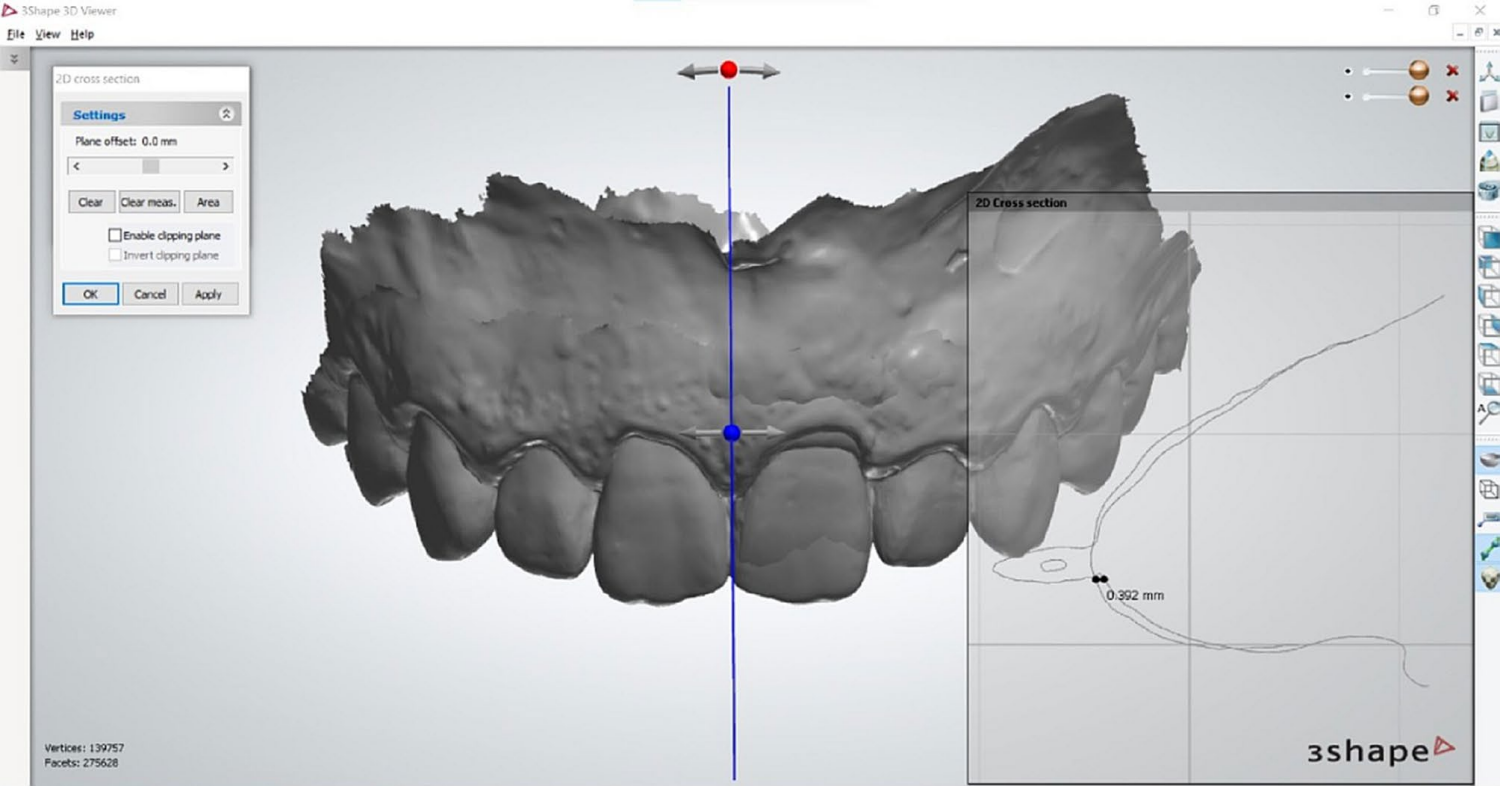
Measuring the soft tissue thickness at the mesial papilla of the central incisor by superimposing the pre- and postoperative scans to standardize the measuring line

The extent of mucosal recession was identified at midfacial gingiva and the crest of the mesial and distal papillae by superimposing the Standard tessellated Language (STL) files of the models obtained via IOS, taken at baseline and 12 months after implant placement (Fig. 4).

The 3D software (NemoSmile Design 3D, Nemotec, Madrid, Spain) aligned the pre-and postoperative models through three identical points identified on their surfaces. The best-fit algorithm of the software perfected the superimposition process. The superimposed models were then imported into an STL viewer (3Shape Ortho viewer, 3Shape, Denmark), where the measurements were performed. This method has been proven accurate for volumetric measurements of hard and soft tissues [27].

Numerical data were explored for normality using the Kolmogorov-Smirnov and Shapiro-Wilk tests. All the data showed a non-normal (non-parametric) distribution. Data are presented as means, standard deviations (SD), medians, and ranges. Since the data were non-parametric, the Mann-Whitney U test was used to compare both groups. We used the Wilcoxon signed-rank test to study changes in time within each group. Qualitative data are presented as frequencies and percentages. The chi-square and Fisher’s exact tests were used to compare the groups. Subgroup analysis was performed according to implant size to examine its effect on bone and soft tissue changes. Unfortunately, subgrouping based on the implant site was impossible as only one premolar and two canine sites were available. To test the reliability of radiographic and soft tissue readings between the two outcome assessors, an interclass reliability test was used. The significance level was set at *P* ≤ 0.05, and statistical analysis was performed using IBM SPSS (Statistics for Windows, version 23.0. IBM Corp, Armonk, NY, USA).

## Results

**Fig. 5:**
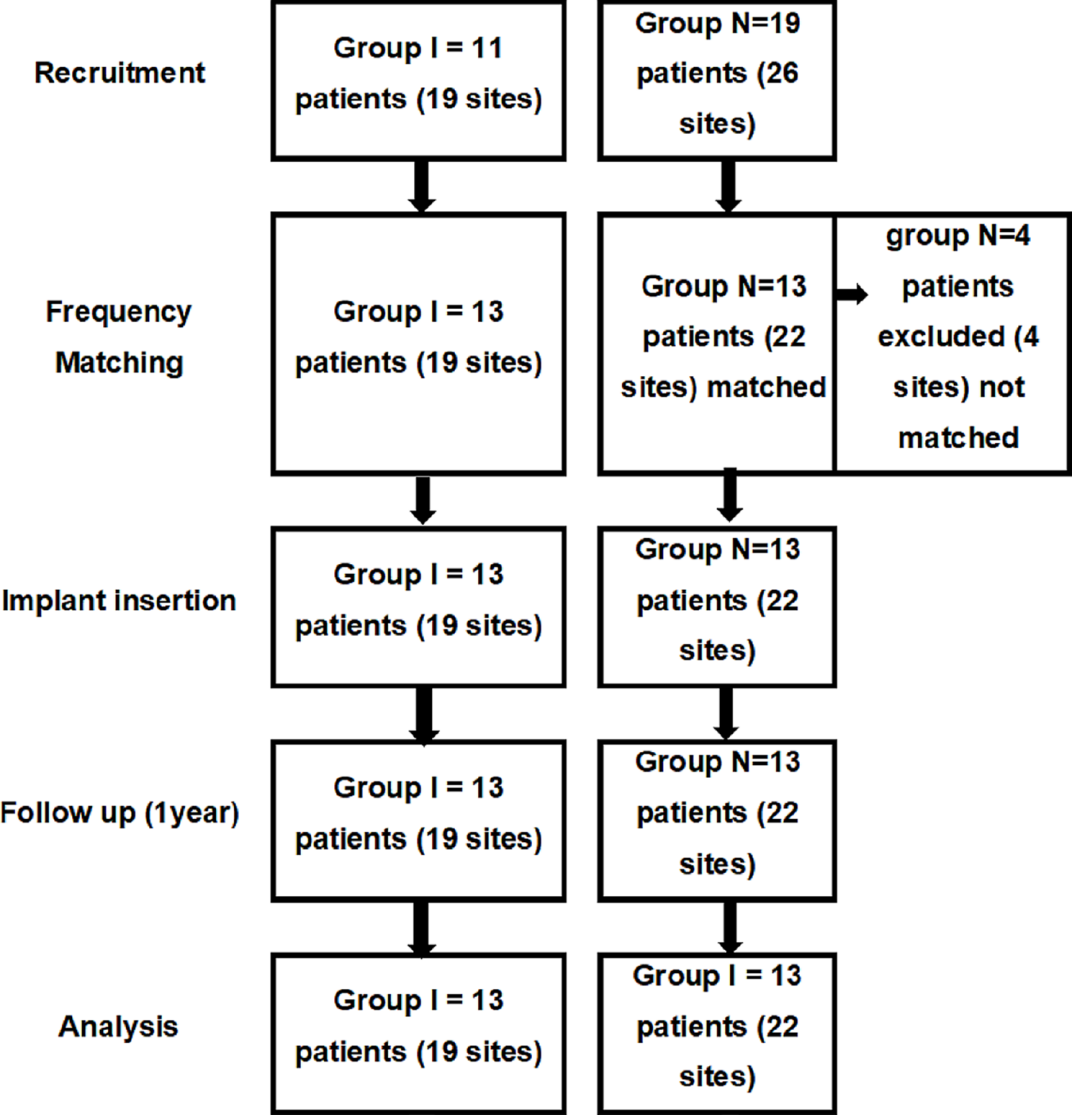
Participants’ flowchart

Despite requiring a sample size of 34 implant sites, 17 per group, to detect significant differences between implant survival of the studied groups, 30 patients were recruited with a total of 45 implant sites. To achieve frequency matching between the groups regarding age and sex in the recruited sample, 4 patients from group N were excluded with 4 implant sites. The 4 excluded patients were all females with an age range of 69–75, a thing that could have worsened the results of the non-infected sockets group and resulted in bias in favor of group I. Finally, a total of 26 patients with 41 implant sites were included, 13 in each group (19 sites were infected and 22 were non-infected). This means that 6 patients in group I and 9 in group N received 2 implants None of the patients were lost to follow up. The number of participants at different stages of the study is shown in Fig. 5.

The most prevalent active sign of infection in group I was sinus tract formation (57.9%), followed by swelling/acute (15.8%), radiographic signs of chronic infection (15.8%), and fistula/chronic infection (10.5%). Both groups showed 100% implant survival. However, the groups differed significantly between implant sizes and site distribution (Table 1). Therefore, the authors decided to do a subgroup analysis to study the effect of the size on the results. Unfortunately, the absence of canines and premolars in the group I made statistical testing for the effect of implant site not possible.

**Table 1:** Base line characteristics of infected and non-infected socket groups

Variable	Infected socket	Non-infected socket	*P*-value
Age [n]			NA
30–40 years	4	4	(matc hed)
41–55 years	9	9	
Gender [n (%)]			
Male	3 (23%)	3 (23%)	NA (matched)
Female	10 (77%)	10 (77%)	
Site [n (%)]			
Central incisor	16 (84.2%)	9 (40.9%)	0.018*
Lateral incisor	3 (15.8%)	10 (45.5%)	
Canine	0 (0%)	2 (9.1%)	
Premolar	0 (0%)	1 (4.5%)	
Implant size [n (%)]			
3.4 mm	9 (47.4%)	17 (77.3%)	0.048*
4.2 mm	10 (52.6%)	5 (22.7%)	

^†^ NA: not applicable; *: Significant at *P* ≤ 0.05

Studying the effect of infection revealed no significant difference between groups N and I regarding apical, mid, and crestal bone thickness and soft tissue level, except at the mesial papilla, where the recession was significantly greater in group N than that in group I (Table 2).

**Table 2:** Descriptive statistics and results of Mann-Whitney U test for comparison between bone thickness, changes in bone thickness (mm) in infected and non-infected groups, Wilcoxon signed-rank test for comparison between pre- and post-operative measurements within each group

Level	Time period	Infected socket (*n* = 19 implants)		Non-infected socket (*n* = 22 implants)		*P*-value	Effect size (d)
		Mean (SD) ^†††^	Median (range)	Mean (SD) ^†††^	Median (range)		
DP^**¶**^	0–12 m^**††**^	-0.34 (0.51)	-0.26 (-1.47-0.42)	-0.74 (1.11)	-0.39 (-4.67-0.42)	0.314	0.318
MFG^**‡‡**^	0–12 m^**††**^	-0.5 (0.87)	-0.39 (-2.46-0.62)	-0.86 (1.19)	-0.77 (-2.87-1.22)	0.229	0.382
MP^**§§**^	0–12 m^**††**^	-0.51 (0.91)	-0.36 (-2.86-0.52)	-1.24 (1.31)	-0.77 (-4.24-0.42)	0.036*	0.691
BTA^**†**^	0 m^**††**^	1.73 (1.86)	0.98 (0-5.62)	1.99 (1.18)	2.01 (0-4.2)	0.169	0.439
	12 m^**††**^	2.76 (1.78)	2.53 (0.81–6.11)	2.42 (0.94)	2.51 (1.12–4.2)	1	0
	0–12 m^**††**^	1.03 (1.6)	0.39 (0-6.11)	0.43 (0.68)	0 (0-2.44)	0.312	0.297
	*P*-value (TC) ^**‡‡‡**^	0.005*		0.005*			
	*Effect size (d)*	1.679		1.492			
BTM^**§**^	0 m^**††**^	0.48 (0.78)	0 (0-3.13)	0.65 (0.67)	0.52 (0-1.74)	0.205	0.382
	12 m^**††**^	2.46 (1.06)	2.24 (1.18–5.2)	2.04 (0.64)	1.82 (1.25–3.32)	0.224	0.387
	0–12 m^**††**^	1.99 (1.03)	2 (0-4.39)	1.39 (1.01)	1.43 (0-3.32)	0.105	0.523
	*P*-value (TC) ^**‡‡‡**^	< 0.001*		< 0.001*			
	*Effect size (d)*	3.283		3.047			
BTC^**‡**^	0 m^**††**^	0.63 (0.68)	0.47 (0-2.01)	0.42 (0.45)	0.41 (0-3.32)	0.447	0.23
	12 m^**††**^	1.91 (0.91)	1.74 (0.73–4.22)	1.95 (0.77)	1.92 (0.73–3.93)	0.865	0.053
	0–12 m^**††**^	1.28 (1.14)	1.01 (-0.41-3.67)	1.53 (0.92)	1.33 (0.09–3.93)	0.278	0.344
	*P*-value (TC) ^**‡‡‡**^	< 0.001*		< 0.001*			
	*Effect size (d)*	3.096		3.633			

^†^ BTA: apical buccal bone thickness, ^‡^ BTC: crestal buccal bone thickness, ^§^BTM: midbuccal bone thickness, ^¶^DP: distal papilla, ^††^m: months, ^‡‡^MFG: midfacial gingiva, ^§§^MP: mesial papilla, ^¶¶^n: number, ^†††^ SD: standard deviation, ^‡‡‡^ TC: time change, *: Significant at P ≤ *0.05*

Changes over time were significant in both groups’ regarding apical, mid, and crestal bone thicknesses (Table 2). Since in this study, mainly anterior teeth were involved, the diameter of the inserted implants were either 3.4–4.2 mm. Subgrouping according to the implants size revealed a significant difference between group I and N in the mesial papilla level and the baseline apical bone thickness of the 3.4 mm diameter implant, favoring group I (Table 3). Results of the interclass reliability of the two assessors revealed an interclass correlation coefficient of 0.92 for the radiographic assessment and of 0.94 for the soft tissue measurements.

**Table 3:** Descriptive statistics and results of Mann-Whitney U test for comparison between bone thickness, changes in bone thickness (mm) in infected and non-infected socket groups

Implant	Level	Time	Infected socket		Non-infected socket		*P*-value	Effect size (d)
			Mean (SD) ^¶¶^	Median (range)	Mean (SD) ^¶¶^	Median (range)		
	DP^**¶**^	0–12 m^**††**^	-0.29 (0.36)	-0.26 (-1.06-0.13)	-0.88 (1.16)	-0.44 (-4.67-0.22)	0.133	0.632
	MFP^**‡‡**^	0–12 m^**††**^	-0.42 (0.51)	-0.4 (-1.11-0.31)	-1.12 (1.09)	-1.1 (-2.87-0.61)	0.085	0.732
	MP^**§§**^	0–12 m^**††**^	-0.42 (0.3)	-0.42 (-0.84-0.25)	-1.47 (1.35)	-0.97 (-4.24-0.42)	0.016*	1.066
3.4 mm	BTA^**†**^	0 m^**††**^	1.05 (1.1)	0.98 (0-3.73)	2.06 (1.16)	2.1 (0-4.2)	0.025*	0.976
		12 m^**††**^	1.85 (1)	1.3 (1.08–3.73)	2.54 (0.85)	2.63 (1.12–4.2)	0.085	0.732
		0–12 m^**††**^	0.8 (0.88)	0.56 (0-2.19)	0.48 (0.75)	0 (0-2.44)	0.426	0.332
	BTM^**§**^	0 m^**††**^	0.27 (0.42)	0 (0-0.96)	0.61 (0.69)	0.37 (0-1.74)	0.241	0.49
		12 m^**††**^	2.21 (0.73)	2.23 (1.18–3.23)	2.18 (0.66)	2.26 (1.25–3.32)	0.916	0.053
		0–12 m^**††**^	1.94 (0.7)	2.07 (0.8–2.9)	1.56 (1.02)	1.55 (0-3.32)	0.396	0.354
	BTC^**‡**^	0 m^**††**^	0.7 (0.78)	0.47 (0-1.68)	0.35 (0.37)	0.41 (0-0.87)	0.426	0.332
		12 m^**††**^	1.77 (0.56)	1.74 (1.01–2.7)	1.87 (0.84)	1.73 (0.73–3.93)	0.874	0.074
		0–12 m^**††**^	1.07 (0.98)	1.18 (-0.41-2.26)	1.52 (0.93)	1.32 (0.09–3.93)	0.560	0.245
4.2 mm	DP^**¶**^ MFG^**‡‡**^ MP^**§§**^	0–12 m^**††**^	-0.38 (0.63)	-0.28 (-1.47-0.42)	-0.27 (0.85)	0 (-1.74-0.42)	0.513	0.353
		0–12 m^**††**^	-0.57 (1.12)	-0.34 (-2.46-0.62)	0.04 (1.16)	0.54 (-1.61-1.22)	0.371	0.523
		0–12 m^**††**^	-0.59 (1.25)	-0.28 (-2.86-0.52)	-0.49 (0.85)	-0.16 (-1.96-0.22)	0.953	0.063
	BTA^**†**^	0 m^**††**^	2.35 (2.22)	1.76 (0-5.62)	1.76 (1.35)	1.13 (0.83–4.03)	0.859	0.127
		12 m^**††**^	3.58 (1.97)	3.84 (0.81–6.11)	2.03 (1.22)	1.61 (1.13–4.13)	0.129	0.861
		0–12 m^**††**^	1.23 (2.08)	0.2 (0-6.11)	0.26 (0.33)	0.1 (0-0.76)	0.768	0.191
	BTM^**§**^	0 m^**††**^	0.66 (0.99)	0.23 (0-3.13)	0.79 (0.67)	0.63 (0-1.66)	0.440	0.42
		12 m^**††**^	2.69 (1.29)	2.3 (1.19–5.2)	1.58 (0.28)	1.64 (1.26–1.97)	0.055	1.125
		0–12 m^**††**^	2.03 (1.29)	1.64 (0-4.39)	0.79 (0.8)	0.74 (0-1.64)	0.129	0.861
	BTC^**‡**^	0 m^**††**^	0.56 (0.61)	0.5 (0-2.01)	0.65 (0.65)	0.7 (0-1.64)	0.679	0.255
		12 m^**††**^	2.04 (1.16)	1.89 (0.73–4.22)	2.21 (0.46)	2.42 (1.55–2.71)	0.513	0.386
		0–12 m^**††**^	1.48 (1.28)	0.67 (0.34–3.67)	1.56 (0.96)	1.33 (0.51–2.71)	0.594	0.32

^†^ BTA: apical buccal bone thickness, ^‡^ BTC: crestal buccal bone thickness, ^§^BTM: midbuccal bone thickness, ^¶^DP: distal papilla, ^††^m: months, ^‡‡^MFG: midfacial gingiva, ^§§^MP: mesial papilla, ^¶¶^SD: standard deviation, ^†††^TC: time change, *: Significant at P ≤ *0.05*

## Discussion

Unrestorable infected teeth are indicated for extraction and are usually replaced by delayed implant placement [28–30]. Several treatment procedures have been recommended, including chemical and mechanical debridement to enhance the prognosis of immediately placed implants in infected sockets [31]. Systemic antibiotics [32], oral rinse [33], guided tissue or bone regeneration [19, 34], the use of plasma rich growth factors (PRGF) [35–37] and immediate restoration were suggested.

Aiuto et al. [38], succeeded in treating infected sites by decontaminating them with an Er, Cr: YSGG laser device followed by immediate implant. However, the study focused mainly on the marginal bone levels (MBL) and radiographic success but did not evaluate the soft tissue levels. Kakar et al. [11], reported similar findings for laser decontamination of the infected sockets and claimed that implant survival rates for these sockets were similar to the non-infected ones, when they were grafted with an in situ hardening alloplastic bone graft material using a non-submerged healing protocol. On the other hand, a one-year study done on 28 patients, presented with infected sites, were treated by immediate placement of a single laser-micro-grooved implants with the adjunct of a highly porous anorganic porcine bone mineral matrix and a collagen wound dressing, showed benefits in maintaining marginal bone levels and soft tissue contour around post-extraction implants in the esthetic zone [39]. However, the majority of patients (18/28) presented with thick gingival biotypes, a variable that might have had an influence on maintaining soft tissue levels.

In our study the 6-day protocol combined with VST was utilized to treat type 2 infected sockets with thin gingival biotype. The protocol eliminates the source of infection by removing the root tip and achieves a sterile socket environment by eradicating the communication between the oral cavity and the socket, that was created by fistulae and/or sinus tracts. This optimized the success of the placed dental implants and bone grafts. Besides, it maintains the socket architecture near to the orifice and preserves the socket topography, thereby providing a safer environment for the VST protocol. However, the protocol was implemented for both infected and non-infected sockets to achieve comparability between the 2 groups and to decrease the risk of performance bias. This resulted in a 100% implant survival, despite beginning with a defective labial bone plate. It also shortened the overall treatment time by up to two-thirds of that required for early or delayed implant placement.

The enhanced bone outcomes presented in this study could be attributed to the slowly biodegradable cortical membrane used in the study. The latter maintained the regenerative bone space and overcame the deleterious remodeling and resorptive effects commonly reported following tooth extraction. Besides, the used bone graft is biocompatible, bioavailable and osteoconductive, where the autogenous bone chips have magnificent osteogenic properties [23]. On the other hand, the xenograft used in the study (Gen-Os® Tecnoss®) contains 22% collagen that gradually resorbs, providing better remodeling than deproteinized bovine bone matrix (DBBM), more new vital bone formation and more vascularization, while preserving the original graft shape and volume [40]. This might explain the significant increase in the facial bone thickness of both groups, one year following implant placement, at the different implant levels compared with the baseline measurements. On the other hand, conventional collagen membranes were reported to have a tendency for rapid biodegradation by the enzymatic activity of macrophages and polymorphonuclear leukocytes, thereby, limiting their ability to preserve bone contour, especially in type II sockets [41, 42].

The 6-day protocol achieved sufficient decontamination, especially in group I sites, making the results of the bone and soft tissue thickness statistically insignificant. The retained natural tooth contour contributed to a lesser mucosal recession in both groups, whereby the generic mean recession value was from 0.29 to 0.51 mm and 0.39–1.47 mm in groups I and N, respectively. Furthermore, VST is a surgical technique that requires no vertical releasing incisions. This maintained the blood supply in groups N and I, regardless of the presence of infection, and hence maintained the marginal soft tissues of both groups similiarly. Since all sockets had thin gingival phenotype, CT graft was placed to compensate for the detrimental effect of this phenotype on bone thickness loss and mucosal recession [43]. Since implant size distribution differed significantly in the 2 groups, the authors decided to do a subgroup analysis to study the effect of the size on the results. No statistically significant difference in bone thickness and mucosal level of the groups was found, regardless of the implant size at all levels of measurements, except for the mesial papilla level in the main comparison and the subgroup analysis, and the apical bone thickness changes of the 3.4 mm diameter implants, all favoring group I. Several authors [8–12, 15–17] agreed with the non-significant findings between infected and non-infected sockets regarding implant survival. Chen et al. [17] added that the non-significant findings were also extended to include soft and hard tissue levels. These authors [8–12, 15–17] added that immediate implant placement in infected sockets is possible, provided that the socket was thoroughly debrided, guided bone regeneration was performed, aggressive antibiotic treatment was prescribed, and primary implant stability was achieved. The observed difference between the mucosal levels of groups N and I in the mesial papilla could be due to chance (α error or random error), where the papilla height is affected by the distance between the contact point and the alveolar crest of the adjacent teeth, the distance between the implant and its adjacent natural teeth, and the anatomy of the teeth [44, 45].

The increased bone thickness observed in both groups, revealed that combing the 6-day protocol with VST achieved similar results in the studied groups, where the generic mean bone thickness gain ranged from 0.85 to 2.4 mm and 0.26–1.63 mm in groups I and N, respectively. Confounders in the study were managed either by frequency matching or by subgroup analysis to decrease the risk of confounding bias. Despite being a prospective cohort study, which eliminates any risk of recall bias or lack of temporality, the study has a relatively short follow up period. The calculated sample size eliminates the risk of β error and decreases the possibility of underpowered study. However, results of subgroup analysis might require a bigger sample size. Its results are only suggestive but no recommendations can be based on this subgroup analysis.

Results of this cohort study are generalizable since the results apply to males and females with type 2 infected and non-infected sockets in the esthetic zone within the age range of 22–60. However, randomized clinical trials with calculated sample sizes and longer follow up periods are still highly recommended to compare immediate implant placement in infected sockets using the 6-day protocol and the VST with delayed implant placement and contour augmentation.

## Conclusion

Within the limitations of this study immediate implant placement in type II infected sockets using the 6-day protocol and VST achieved 100% implant survival, while maintaining the regenerated facial bone thickness with minimal mucosal recession. However, we recommend further randomized studies with a larger sample size to monitor hard and soft tissue changes over a longer follow-up period.

## Data Availability

The datasets used and/or analyzed during the current study are available from the corresponding author on reasonable request.

## Abbreviations

3D: 3 dimensional
CBCT: Cone beam computed tomography
Cm: Centimeter
CT: Connective tissue graft
DDBM: Dried demineralized bone matrix
Group I: Patients with infected sockets
Group N: Patients with non-infected sockets
h: Hours
KVp: Kilovoltage peak
mg: Milligram
ml: Milliliter
mm: Millimeter
PRGF: Plasma rich growth factors
SD: Standard deviation
STL: Standard Tessellated Language
STROBE: Strengthening the Reporting of Observational Studies in Epidemiology
VST: Vestibular socket therapy
